# A study of post‐traumatic stress disorder in schizophrenic patients 35 years after experiencing the Tangshan earthquake

**DOI:** 10.1002/brb3.2963

**Published:** 2023-04-17

**Authors:** Wenyou Ma, Rong Lei, Yan Sun, Xiaoliang Liang, Shun Zhang, Changqi Wang, Ying Tang, Gang Wang, Hebin Li, Zhenjian Yu

**Affiliations:** ^1^ Department of Clinical Psychology Kailuan Mental Health Center Affiliated to North China University of Science and Technology Tangshan Hebei China; ^2^ Department of Children and Adolescents Second People's Hospital of Huizhou Huizhou China

**Keywords:** dissociative experience, earthquake, post‐traumatic stress disorder, schizophrenia

## Abstract

**Background:**

This study aimed to investigate the relationship between dissociative experiences, post‐traumatic stress disorder (PTSD), and psychiatric symptoms exhibited by schizophrenic patients 35 years after the Tangshan earthquake.

**Methods:**

Seventy‐one schizophrenic patients who had experienced the Tangshan earthquake were selected and evaluated by the Post‐traumatic Dissociative Experience Questionnaire (PDEQ), thPTSD Checklist‐Civilian Version (PCL‐C), and the Positive and Negative Symptom Scale (PANSS).

**Results:**

The score of Group B (re‐experiencing symptoms) in PCL‐C was significantly positively correlated with age and significantly negatively correlated with the course of schizophrenia. Both gender and marriage were significantly positively correlated with the score of PCL‐C Group D (irritability symptoms caused by hyperarousal). The PDEQ score was negatively correlated with thecourse of schizophrenia and positively correlated with the presence of sleep difficulties. Significant differences were found between the PCL‐C scores of the positive and negative symptoms of the three core symptom groups; the positive rate of Group B was significantly higher than that of Group D, and the positive rate of Group C was significantly higher than that of Group D. The PCL‐C total score was positively correlated with the negative symptom factor score of PANSS; Group C's symptoms were significantly negativelycorrelated with the positive factor score of PNASS; andGroup D's‘ symptoms were significantly negatively correlated with the PANSS total score and the positive factor score.

**Conclusion:**

When consiering patients with schizophrenia post the Tangshan earthquake, age, gender, and marital status were all positively correlated with PTSD. The course of schizophrenia was negatively associated with PTSD and dissociative experiences. PTSD was positively correlated with the negative symptoms of schizophrenia and negatively correlated with the positive symptoms of schizophrenia. Thus, the conditions and symptoms of PTSD may interact with those of schizophrenia.

## Graphical abstract

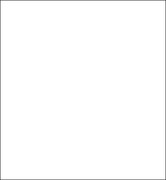

## 1 INTRODUCTION

Research has revealed that over 69.5‐98% of the word population has experienced a traumatic event in their lifetime (Gearon et al., 2003). In addition to the high economic costs and physical damage, natural disasters such as earthquakes, hurricanes, tornadoes, floods, and fires can lead to depression, schizophrenia, and a range of other emotional and physical health problems (Baryshnikova & Pham, 2019). Natural disasters occur without warning and do not allow people the opportunity to prepare psychologically, resulting in emotional effects ranging from depression to dissociative symptoms; the most notable among these is post‐traumatic stress disorder (PTSD) (Jia et al., 2015; Wang et al., 2015). PTSD is a type of psychiatric disorder that can occur when an individual is exposed to death, serious injury, or threats to physical safety. Symptoms may appear and persist long after the traumatic event has occurred. PTSD is characterized by three core groups of symptoms: traumatic reexperiencing, avoidance and numbing, and heightened alertness (Armour et al., 2019; Compean & Hamner, 2019). However, PTSD is not the only diagnosis for those who have experienced a traumatic event (Auxemery, 2018). Other outcomes associated with trauma include anxiety, depression, dissociative experiences, and psychiatric symptoms (Auxéméry, 2015; Baryshnikova & Pham, 2019; Bickel et al., 2020; Lin et al., 2016; Roley et al., 2015; Wang et al., 2020). Therefore, it is essential to understand the relationship between these symptom clusters and mental distress to better treat those affected by traumatic events.

PTSD can lead to relapse and deterioration of mental illness, heightened substance abuse, and increased service costs. Studies have found that the incidence of PTSD among those with schizophrenia is approximately 13–29% (Mueser et al., 1998; Resnick et al., 2003), while the lifetime prevalence of PTSD in the general population is only 7.8–9.2% (Kessler et al., 1995; Stein et al., 1997). Studies have also demonstrated a strong correlation between post‐traumatic dissociative experiences and PTSD in patients with schizophrenia; however, the precise relationship between these three conditions remains unclear. Two primary theories have been proposed in this regard: post‐traumatic dissociative symptoms may serve as a protective measure against post‐traumatic distress (Van der Kolk & Van der Hart, 1989), or they may be a result of severe post‐traumatic distress (Bernat et al., 1998; Friedman, 2000). Further research is needed to determine the relationships between PTSD, post‐traumatic dissociative symptoms, and psychiatric symptoms.

In the present study, a preliminary investigation was conducted into the relationship between dissociative experience, PTSD, and psychiatric symptoms in schizophrenic patients 35 years after experiencing the Tangshan earthquake. The aim was  to determine the effects of trauma on people with schizophrenia and to gain insights into the links between dissociative experience, PTSD, and mental distress.

## 2 METHODS

### 2.1 Study participants

The research protocol for this study was approved by the Ethics Committee of the Kailuan Mental Health Center affiliated to North China University of Science and Technology. Patients who experienced the Tangshan earthquake in 1976, were later diagnosed with schizophrenia, and were hospitalized at the Kailuan Mental Health Center affiliated to North China University of Technology in 2011 were selected as the research subjects. The inclusion criteria were as follows: first‐time experience of an earthquake ; no neurological diseases, such as epilepsy, cerebrovascular accident, or orgainic brain diseases; no history of heavy drinking; and diagnosed with schizophrenia according to the International Classification of Diseases‐10 (ICD‐10). A total of 71 patients were enrolled; 59 were male and 12 were female. Informed consent for this study was obtained from all patients or their families.

### 2.2 General investigation

The demographic and clinical data of 71 hospitalized patients were collected, which included gender, age, occupation, educational level, years of education, course of the disease, and other information.

### 2.3 Scales

The Peritraumatic Dissociative Experiences Questionnaire (PDEQ) was designed by Professor Marmar in the United States and has been used to investigate the dissociative symptoms of persons exposed to traumatic events (Fikretoglu et al., 2006). It is currently the most widely used dissociative experience assessment tool in trauma‐related research. The questionnaire consist of 10 items that are scored on a scale of 0 to 4 according to severity. Research has shown that this questionnaire has good reliability, validity, and certain operability (Birmes et al., 2005). In the present study, the PDEQ was used to evaluate the dissociative symptoms exhibited by schizophrenia patients after an earthquake.

The PTSD Checklist‐Civilian Version (PCL‐C) has proven to be reliable and valid for evaluating of the symptoms and severity of PTSD. The PCL‐C includes 17 items related to three groups of symptoms: Group B (re‐experiencing symptoms) consists of five items, Group C (emotional numbing and avoidance symptoms) consists of seven items, and Group D (irritability symptoms caused by hyperarousal) consisits of five items. Each item is rated on a scale of 1 to 5 in the order of asymptomatic, mild, moderate, severe, and very severe. The scores of the individual items are added to obtain the total score, and the higher the total score, the greater the likelihood of PTSD. At the same time, if even one of the five items in Group B has a score greater than 2, Group B is considered positive; if three or more of the seven items in Group C have scores greater than 2, Group C is considered positive; and if two or more of the five items in Group D score more than 2 , Group D is judged as positive.

The Positive and Negative Symptom Scale (PANSS) consis of 30 items, including seven positive scale items, seven negative scale items, 16 general psychopathology scale items, and three supplementary items that assess the risk of aggression. The total PANSS score, the positive factor (P factor) score, the negative factor (N factor) score, and the general psychopathology scale (G factor) score can be calculated separately. Each item in the PANSS has its own definition and a specific seven‐level operability scoring standard. The scale has good reliability and validity and can be used to evaluate positive and negative symptoms in patients with schizophrenia.

### 2.4 Scale evaluation

The patients were asked to recall their feelings and mental states after the earthquake, and their poor cognitive status was evaluated. Following this, the PDEQ, PCL‐C, and PANSS were assessed.

### 2.5 Statistical analysis

The statistical analysis was performed using SPSS 22.0 software. The Student's *t*‐test and the Wilcoxon rank‐sum test were used to compare the measurement data that were expressed as mean ± standard deviation (*X* ± *s*). The chi‐square test was used to analyze the difference between the count data of the two groups; the count data were expressed as percentages. Pearson's correlation analysis was used to determine the correlation between any two numerical variables, and Spearman's correlation analysis was used to determine the correlations among the rank variables. *p* < .05 indicated that the difference was statistically significant.

## 3 RESULTS

### 3.1 Patients’ general information

The average age of the patients, including 59 males (83.1%) and 12 females (16.9%), was 60.87 ± 7.44 years. There were 9 cases involving underground workers (12.7%), 59 cases involving surfacemen (83.1%), and 3 cases involving cadres (4.2%). There were 11 (15.5%) cases with a positive family history of psychosis, and 60 (84.5%) with a negative family history. With regard to marital status, 27 cases (38.0%) were unmarried, 19 (26.8%) were married, 21 (29.6%) were divorced, and 4 (5.6%) were widowed. The course of schizophrenia was 20.80 ± 9.02 years, the patient education was 8.31 ± 1.87 years, and the number of family members was 2.69 ± 1.42. Regarding the injuries sustained after the earthquake, 39 people (54.9%) were not buried, and 32 (45.1%) were buried; the duration for which they were buried was 1.26 ± 2.46 hours. Among these, 66 people (93.0%) were not unconscious, and 5 (7.0%) were unconscious.

### 3.2 The effect of being buried or not

In relation to the earthquake experience, the *t*‐test analysis(Table 1) showed that the participants’ schizophrenia course, dissociative experience, PTSD, and PANSS score were not significantly different from the participants’ experience of whether they were buried in the earthquake (*p* > .05). Table 2 presents the correlations between the burial time and the participants’ scores in each of the above surveys. The results showed that burial time did not have any effect on dissociative experiences, PTSD, or other mental reactions.

**TABLE 1:** The impact of whether the participant was buried in the earthquake on scales’ valuations

Variable	Exposed participants (*n* = 39)	Buried participants (*n* = 32)	*t* Test	*p* Value
Schizophrenia course	21.74 ± 9.01	19.66 ± 9.05	0.969	.336
Score of PDEQ	15.13 ± 6.96	14.53 ± 5.84	0.386	.701
Score of PCL‐C	30.64 ± 7.35	28.50 ± 7.95	1.175	.244
Score of PANSS	68.71 ± 22.06	66.45 ± 14.84	0.472	.639

PDEQ: Peritraumatic Dissociative Experiences Questionnaire; PCL‐C: Post‐Ttraumatic Stress Disorder Checklist‐Civilian Version; PANSS: Positive and Negative Symptom Scale.

**TABLE 2:** Pearson's correlations between burial time and scales’ evaluations

Variables	Schizophrenia course	Score of PDEQ	Score of PCL‐C	Score of PANSS
Burial time	−0.089	0.064	−0.047	0.064
*p* Value	.459	.599	.698	.618

PDEQ: Peritraumatic Dissociative Experiences Questionnaire; PCL‐C: Post‐Traumatic Stress Disorder Checklist‐Civilian Version; PANSS: Positive and Negative Symptom Scale.

### 3.3 Correlation between core symptoms of PCL‐C and participants’ background characteristics

There were no significant differences in gender, age, occupation, years of education, and duration of schizophrenia among patients grouped according to the three groups of syndroms in the PCL‐C (*p* >  .05).

There was a significant positive correlation between age and the score of Group B (re‐experiencing symptoms) (*r* = 0.242, *p* <.05). However, the course of schizophrenia was negatively correlated with the re‐experiencing symptoms (*r* = −0.242, *p* < .05). Other background characteristics did not manifest significant associations with the score of Group B (*p* > .05). Furthermore, the emotional numbing and avoidance symptoms (Group C) were not associated with any background characteristics. Spearman's analysis showed that gender (*r* = 0.273, *p* < .05) and marital characteristics (*r* = 0.268, *p* < .05) were significantly positively correlated with the score of Group D (irritability symptoms caused by hyperarousal), while the remaining factors were not significantly correlated with the score of Group D (Table 3).

**TABLE 3:** Correlation analysis between three core symptoms in post‐traumatic stress disorder checklist‐civilian version (PCL‐C) and participants’ background characteristics

Variable	Score ofGroup B	Score of Group C	Score of Group D
Age	0.242^*^	0.116	0.214
Education years	−0.108	−0.049	−0.109
Schizophrenia course	−0.242^*^	−0.002	−0.230^a^
Gender	0.206	0.040	0.273^*^
Occupation	−0.108	−0.128	0.018
Marriage	0.138	0.107	0.268^*^
Education	−0.202	−0.096	0.167

^*^ *p* < .05.

^a^ *p* ≈ .05.

### 3.4 Correlation between post‐traumatic dissociative experience and PTSD, schizophrenia course, and sleep difficulties

Correlation analyses were conducted separately with the scores on subscales to investigate whether post‐traumatic dissociative experiences were related to schizophrenia, PTSD, and sleep difficulties. Dissociative experience was found to be significantly negatively correlated with the course of schizophrenia (20.80 ± 9.02 years) (*r* = −0.240, *p* = .044), and significantly positively correlated with the presence of sleep difficulties (*r* = 0.296, *p* = .012). The data results are presented in Table 4.

**TABLE 4:** Correlation analysis between PDEQ score and PTSD, schizophrenia course, and sleep difficulties

Variable	Score of PCL‐C	Schizophrenia course	PTSD cognition	Sleep difficulties
Score of PDEQ	0.224	−0.240	0.235	0.296
*p* Value	.061	.044	.073	.012

PTSD: post‐traumatic stress disorder; PCL‐C: PTSD Checklist‐Civilian Version; PDEQ: Peritraumatic Dissociative Experiences Questionnaire.

### 3.5 The detection of PTSD symptoms

Considering the PCL‐C scoring criteria, the diagnosis of PTSD was confirmed in only 2 (2.82%) of the 71 patients.

Regarding the detection of PTSD symptoms in Table 5, 55 cases (77.46%) were positive for re‐experiencing symptoms (Group B), and 16 cases (22.54%) were negative; emotional numbing and avoidance symptoms (Group C) were positive in 48 cases (67.61%) and negative in 23 cases (32.39%); irritability symptoms caused by hyperarousal (Group D) were positive in 33 cases (46.48%) and negative in 38 cases (53.52%). We witnessed significant differences in the three groups' results between the PCL‐C scores of the positive and negative symptoms of PTSD (all *p* < .05). We also found significant differences in the positive rate between Group B and Group D and between Group C and Group D .

**TABLE 5:** The detection of PTSD

Variable	Number (%)	Score of PCL‐C	*p*0	*p*1
Group B **(+)**	55(77.46%)	31.05 ± 7.54	.004	.188^a^
Group B **(–)**	16(22.54%)	24.94 ± 6.18		
Group C **(+)**	48(67.61%)	32.94 ± 6.87	.000	.000^b^
Group C **(–)**	23(32.39%)	22.87 ± 3.79		
Group D **(+)**	33(46.48%)	34.36 ± 7.60	.000	.011^c^
Group D **(–)**	38(53.52%)	25.61 ± 4.95		

*p*0 is *p* value of the comparison of PCL‐C scores between the positive and negative groups. *p*1 is *p* value of the positive rate comparison for each group.

^a^ *p* Value when Group B was compared with Group C.

^b^ *p* Value when Group B was compared with Group D.

^c^ *p* Value when Group C was compared with Group D.

PTSD: post‐traumatic stress disorder; PCL‐C: PTSD Checklist‐Civilian Version.

### 3.6 Correlations of dissociative experiences and PTSD with schizophrenia symptoms

Participants were assessed for schizophrenia symptoms by employing the PANSS, and the results were as follows: (1) the tota score of PANSS was 67.69 ± 19.02 points; (2) the P factor score was 12.86 ± 5.01 points; (3) the N factor score was 20.00 ± 8.14 points; and (4) the G factor score was 30.71 ± 8.40 points. The correlation analysis of the various scores of all items revealed that PTSD (the PCL‐C score) was strongly correlated with the negative symptoms of schizophrenia (the score of N factor) (*r* = 0.315, *p* = .011). A high level of negative correlation was found between the symptoms of Group C in PCL‐C and P factor score (*r* = −0.296, *p* = .017). The symptoms of Group D were significantly negatively correlated withthe PANSS sum score (*r* = −0.263, *p* = .036) and P factor score (*r* = −0.315, *p* = .041) as shown in Table 6.

**TABLE 6:** Correlation analysis of PDEQ score, PCL‐C total score, and subgroup scores with PANSS total score and factor scores

Variable	Total score of PANSS	Score of P factor	Score of N factor	Score of G factor
PDEQ	−0.223	−1.141	−0.212	−0.140
PCL‐ C	0.185	0.123	0.315	0.085
Group B	0.194	0.001	0.062	0.185
Group C	−0.237	−0.296	−0.202	−0.140
Group D	−0.263	−0.255	−0.198	−0.207
*p*1	.076	.262	.091	.268
*p*2	.143	.329	.011	.500
*p*3	.124	.994	.622	.140
*p*4	.059	.017	.107	.267
*p*5	.036	.041	.114	.098

*p*1 is *p* values obtained from correlation analysis of PDEQ score with PANSS total score and factor scores. *p*2 is *p* values obtained from correlation analysis of PCL‐ C total score with PANSS total score and factor scores. *p*3 is *p* values obtained from correlation analysis of the symptoms of Group B with PANSS total score and factor scores. *p*4 is *p* values obtained from correlation analysis of the symptoms of Group C with PANSS total score and factor scores. *p*5 is *p* values obtained from correlation analysis of the symptoms of Group D with PANSS total score and factor scores.

## 4 DISCUSSION

Earthquakes are natural disasters that cause tremendous physical and psychological damage to people. The mental adjustment applied by different individuals causes them toexperience different levels of stress and behave accordingly. In this study, a preliminary investigation was conducted into the dissociative experiences, PTSD, and mental symptoms of patients with schizophrenia 35 years after the Tangshan earthquake. The research was performed in an outpatient clinical setting and assessed using subscales, followed by a comparison of the various scores for all items.

As shown in Table 1, no differences in the course and severity of schizophrenia were seen between patients who were buried and those who were not, suggesting that being buried during the earthquake has no effect on the course and severity of schizophrenia. Likewise, no difference in the total score for dissociative experience and the total score for PTSD was found between patients who were buried and those who were not, indicating that being buried during the earthquake has no significant effect on dissociative experiences and PTSD. Moreover, as shown inTable 2, there was no significant correlation between the time of burial and the duration of schizophrenia, the total score of PANSS, the total score of PDEQ, and the total score of PTSD, indicating that the time of earthquake burial has no significant effect on schizophrenia, dissociative experiences, and PTSD. As seen in Table 3, age was positively correlated with the score of re‐experiencing symptoms of PTSD, indicating that the older a person's age, the greater traumatic experience, and the younger the a person's age, the less the traumatic experience. This is consistent with previous studies showing that the age of exposure to trauma is correlated with the prevalence and severity of PTSD (Roques et al., 2019). We also showed that the course of schizophrenia was negatively correlated with the re‐experiencing symptoms. Sar et al. (2010) found childhood trauma to be associated with concurrent dissociation in patients with schizophrenia.In this study, hyperarousal was found to be positively associated with gender and marriage, suggesting that men may be more prone to irritability symptoms than women, and those experiencing marital changes, such as divorce, remarriage, and widowhood, are prone to irritability symptoms. Post‐traumatic gender differences have been demonstrated, suggesting that gender may be a biological variable in the occurrence of PTSD and that gender differences in the endocrine system may contribute to the occurrence and development of PTSD (Seligowski et al., 2020). However, no significant correlates were found for emotional numbing and avoidance symptoms in the present study. As shown in Table 4, the PDEQ score was significantly negatively correlated with the duration of schizophrenia and significantly positively correlated with sleep difficulties, indicating thatthe longer the duration of schizophrenia, the lighter the dissociative experiences, and the heavier the sleep difficulties; the shorter the duration of schizophrenia, the heavier the dissociative experiences, and the lighter the sleep disturbance. In a related study, it was found that people experiencing traumatic events have sleep problems and electroencephalogram alterations (Laxminarayan et al., 2020). As seen in Table 5, nearly half of the patients with schizophrenia in the present study showed symptoms of re‐experiencing, hyperarousal, and emotional numbing and avoidance. Thety were most likely to have re‐experiencing symptoms and emotional numbing and avoidance symptoms, followed by hyperarousal symptoms. As seen in Table 6, the PCL‐ C total score was significantly and positively correlated with the negative symptom factor in PANSS, indicating that the more severe the PTSD condition, the more severe the negative symptoms of schizophrenic patients and the more likely the patients are to decline. In a 25‐year follow‐up study, it was found that trauma symptoms and depressive symptoms can persist for decades (Goenjian et al., 2021), often with the diagnosis of PTSD comorbid depression. The emotional numbing and avoidance symptoms of Group C in the PCL‐ C were significantly negatively correlated with the P‐factor score of the PANSS, indicating that the less severe the emotional numbing and avoidance symptoms of PTSD, the more severe the positive symptoms of schizophrenia, and the more severe the emotional numbing and avoidance symptoms, the less severe the positive symptoms of schizophrenia. Group D symptoms in the PCL‐ C were significantly negatively correlated with the total score of PANSS scale (*r* = –0.263, *p* = .036), indicating that the greater the irritability symptoms caused by PTSD hyperarousal, the less severe the schizophrenia; the lesser the irritability symptoms caused by PTSD hyperarousal, the more severe the schizophrenia. Group D symptoms in the PCL‐ C were significantly negatively correlated with the P factor score of PANSS, indicating that the greater the irritability symptoms caused by PTSD hyperarousal, the lesser the positive symptoms of schizophrenia; the lesser the irritability symptoms caused by PTSD hyperarousal, the more severe the positive symptoms of schizophrenia.

According to the scoring criteria of the PCL‐C, two (2.82%) of the 71 schizophrenic patients had co‐morbid PTSD(. Although most schizophrenic patients who experienced the Tangshan earthquake did not have comorbid PTSD 35 years later, they exhibited positive symptoms of PTSD. Earthquakes are natural disasters that cause great physical and psychological damage to people. Mental stress causes individuals to exhibit different degrees of stress, and intense mental stress can easily lead to mental disorders., PTSD is one of the more typical mental disorders. Furthermore, earthquakes also cause unusual personality traits and other psychosomatic problems (Yu et al., 2008). The diagnosis of PTSD based on the diagnostic criteria is relatively easy; however, some people who do not meet the diagnostic criteria still have positive symptoms or other manifestations of PTSD (Auxéméry, 2018). In a study investigating the prevalence of PTSD in 260 orphans 30 years after the Tangshan earthquake (Zhang et al., 2008), 32 (12.3%) orphans still suffered from PTSD. Our findings support the “interaction hypothesis” proposed by Mueser et al. (2002), which suggests that PTSD has both a direct and an indirect influence on mental illness. This hypothesis was also confirmed by Spitzer et al‘s study, which showed that patients with severe mental illness and PTSD symptoms exhibits increased psychopathological distress and autistic features compared with those without PTSD symptoms (Spitzer et al., 2007).

The pathophysiological mechanisms of schizophrenia and PTSD are complex, multifaceted, and not yet fully understood. However, there appears to be some overlap between them. It has been suggested that dysregulation of the hypothalamic‐pituitary‐adrenal (HPA) axis, which regulates the body's stress response, may lead to symptoms of PTSD and schizophrenia via the production of abnormal cortisol levels (Dunlop & Wong, 2019; Mikulska et al., 2021). Furthermore, both disorders are associated with several morphological alterations in the brain, such as abnormalities in the amygdala, hippocampus, and prefrontal cortex (Harnett et al., 2020; Tendilla‐Beltrán et al., 2019). They also exhibit similar neurotransmitter imbalances, involving dopamine, serotonin, and norepinephrine (Bandelow et al., 2017; Joyce, 1993). Autonomic nervous system dysfunction may contribute to both PTSD and schizophrenia, potentially impacting the body's immune system and lessening its ability to fight off infection or illness (Morris & Rao, 2013; Stogios et al., 2021). Moreover, there is evidence that abnormalities in the body's inflammatory response may play a role in the pathogenesis of both PTSD and schizophrenia (Khandaker et al., 2015; Lee et al., 2022). Additionally, both disorders are believed to be caused by a combination of multiple genetic and environmental factors (Smoller, 2016; Zamanpoor, 2020). Further research is needed to gain a fuller understanding of the intrinsic link between these two disorders.

### 4.1 Limitations

As this was a cross‐sectional study, there may have been recall bias when investigating people who had experienced traumatic events. Another limitation is the relatively small sample size of the study and the lack of research on PTSD in a large sample of different genders with schizophrenia. Our results stemmed form a preliminary study on the relationship between the dissociative symptoms, PTSD, and psychiatric symptoms of patients suffering from schizophrenia after the earthquake. A longitudinal study should be conducted in the future to extend the causal relationships identified.

## 5 CONCLUSION

In this study, age, gender, and marital status were all found to have an positive association with traumatic events and PTSD symptoms in schizophrenic patients 35 years after the Tangshan earthquake. Furthermore, the schizophrenia course was negatively correlated with PTSD and dissociative experiences. PTSD was positively correlated with the negative symptoms of schizophrenia and negatively correlated with the positive symptoms of schizophrenia. That means PTSD can have an effect on schizophrenic conditions and positive or negative symptoms; similarly, schizophrenic conditions and positive or negative symptoms can influence PTSD. Ultimately, both schizophrenia and PTSD appear to be complex responses to traumatic events.

## CONFLICT OF INTEREST STATEMENT

All authors have no potential conflict of interest.

## FUNDING INFORMATION

None.

### PEER REVIEW

The peer review history for this article is available at https://publons.com/publon/10.1002/brb3.2963.

## Data Availability

The data that support the findings of this study are available from the corresponding author upon reasonable request.
